# Direct Air Capture Using Electrochemically Regenerated Anion Exchange Resins

**DOI:** 10.1021/acs.est.2c01944

**Published:** 2022-08-04

**Authors:** Qingdian Shu, Marina Haug, Michele Tedesco, Philipp Kuntke, Hubertus V. M. Hamelers

**Affiliations:** † Wetsus, European Centre of Excellence for Sustainable Water Technology, Oostergoweg 9, 8911MA Leeuwarden, The Netherlands; ‡ Environmental Technology, Wageningen University, Bornse Weilanden 9, 6708WG Wageningen, The Netherlands; § Faculty of Natural and Environmental Sciences, Hochschule Zittau/Görlitz, Theodor-Körner-Allee 16, 02763 Zittau, Germany

**Keywords:** carbon capture, amine-functionalized resins, electrochemical cell, pH swing, CO_2_ capture capacity

## Abstract

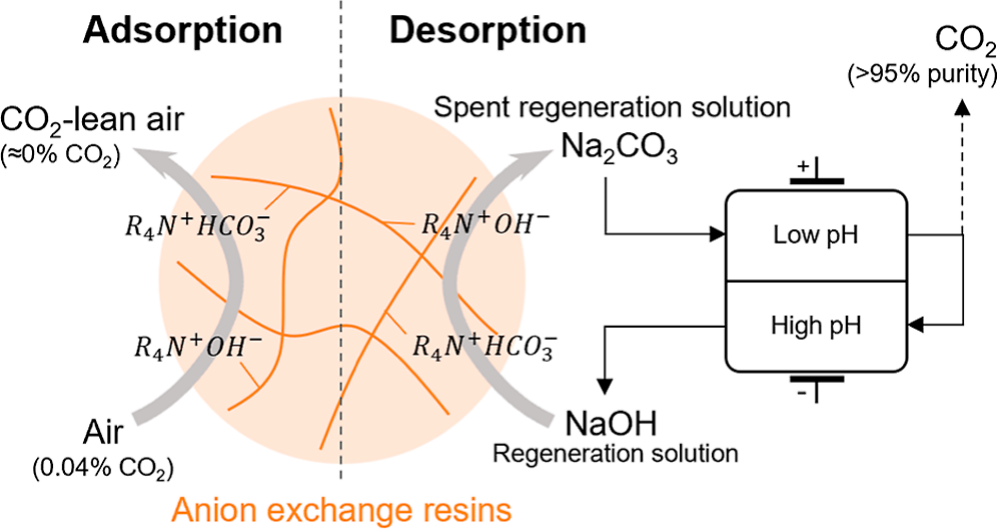

Direct air capture (DAC) aims to remove CO_2_ directly from the atmosphere. In this study, we have demonstrated proof-of-concept of a DAC process combining CO_2_ adsorption in a packed bed of amine-functionalized anion exchange resins (AERs) with a pH swing regeneration using an electrochemical cell (EC). The resin bed was regenerated using the alkaline solution produced in the cathodic compartment of the EC, while high purity CO_2_ (>95%) was desorbed in the acidifying compartment. After regenerating the AERs, some alkaline solution remained on the surface of the resins and provided additional CO_2_ capture capacity during adsorption. The highest CO_2_ capture capacity measured was 1.76 mmol·g^–1^ dry resins. Moreover, as the whole process was operated at room temperature, the resins did not show any apparent degradation after 150 cycles of adsorption–desorption. Furthermore, when the relative humidity of the air source increased from 33 to 84%, the water loss of the process decreased by 63%, while CO_2_ capture capacity fell 22%. Finally, although the pressure drop of the adsorption column (5 ± 1 kPa) and the energy consumption of the EC (537 ± 33 kJ·mol^–1^ at 20 mA·cm^–2^) are high, we have discussed the potential improvements toward a successful upscaling.

## Abstract 2

CO_2_ adsorption using anion exchange resins for direct air capture was successfully integrated with an electrochemical regeneration process.

## 1 Introduction

The atmospheric carbon dioxide (CO_2_) concentration has increased by nearly 50% compared to preindustrial levels.^1^ This increase is primarily ascribed to anthropogenic activities and has caused rapid climate change in recent years.^2−5^ Several carbon capture and storage (CCS) technologies have been proposed and implemented to reduce CO_2_ emissions from point sources.^6−8^ Moreover, carbon dioxide removal (CDR), aiming to remove CO_2_ already in the atmosphere, is an indispensable complement to CCS so that the 1.5 °C targets from Paris Agreement can be achieved.^9−12^ Among the proposed CDR technologies, direct air capture (DAC) has the advantages of capturing CO_2_ from distributed sources and has high flexibility in its deployment location.^13,14^ Despite the relatively high energy costs and materials requirement of current technologies, the development and deployment of DAC technologies are crucial for realizing the Paris Agreement climate goals.^15^

DAC with amine-based sorbents has been extensively studied.^16−19^ Among these studies, amine-functionalized anion exchange resins (AERs), polymeric materials commonly used in desalination and water treatment, have been demonstrated to be eligible candidates with a high CO_2_ capture capacity and low heat capacity.^20−23^ Several methods have been proposed to regenerate the resins after adsorption. For instance, in a temperature swing process with (primary) amine-functionalized AERs, CO_2_ from the air is adsorbed by forming carbamate species, and CO_2_ is then desorbed by heating the resins during regeneration.^20^ Alternatively, in a moisture swing process with (quaternary) amine-functionalized AERs, CO_2_ can be adsorbed by dry resins and desorbed when the resins are wet.^23^ In this case, the dry resins have hydroxide (OH^–^) or carbonate (CO_3_^2–^) as counter-ions of the amine groups that combine with CO_2_ to form bicarbonate (HCO_3_^–^). The amine groups in HCO_3_^–^ form shift to CO_3_^2–^ form and release CO_2_ gas when moisture is added during the desorption step.^23^ Both temperature swing and moisture swing have limitations. The resins are likely to degrade under the high temperature of desorption during temperature swing, and the process requires heat as the energy input, limiting the selection of sustainable energy sources.^24^ Moisture swing is limited by the low CO_2_ capture capacity with high humidity air.^25^ Therefore, although amine-functionalized AERs are competent sorbents for DAC, there is room for a novel regeneration strategy that is benign for the resins and has a low energy input and CO_2_ production at higher pressure.

In desalination and water treatment applications, AERs are commonly regenerated using alkaline solutions. For DAC with quaternary amine-functionalized AERs in OH^–^ form, HCO_3_^–^ and CO_3_^2–^ are formed during the adsorption step according to eqs 1 and 2.^23^
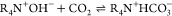
1
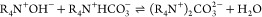
2

In principle, when an alkaline solution is in contact with the resins, the adsorbed HCO_3_^–^ and CO_3_^2–^ are exchanged with OH^–^ in solution, and the resins are regenerated. After regeneration, the resins are in OH^–^ form and the spent regeneration solution has a high concentration of HCO_3_^–^ and CO_3_^2–^. Finally, a desorption process is required to desorb CO_2_ from the spent regeneration solution and reproduce the alkaline solution.

Recently, we developed an electrochemical system that can regenerate spent alkaline absorbents from DAC and desorb high-purity CO_2_ gas under atmospheric pressure.^26^ The electrochemical system uses a pH swing to regenerate the solution in two adjacent compartments separated by a cation exchange membrane (CEM). At low pH, the CO_2_ equilibria displace toward the formation of carbonic acid (H_2_CO_3_*) due to the H^+^ production at the anode. When the chemical potential of oversaturated H_2_CO_3_* is higher than the partial pressure of CO_2_ in the gas phase, CO_2_ can be desorbed. At high pH, the alkaline solution used for regenerating the resins is regenerated due to the OH^–^ production at the cathode.

In this work, we propose a novel process for DAC by combining the adsorption step with AERs and the regeneration step with the electrochemical system. The process operates at room temperature and only uses electricity as energy input. Moreover, the captured CO_2_ is desorbed as high purity CO_2_ gas at atmospheric pressure. The repeatability of the process and stability of the resins were tested by using ambient air as feed. Moreover, air with different humidity values has been investigated to show the impact of humidity on the performance of the process. Finally, we have discussed the application of the technology in terms of energy consumption and the perspectives regarding further developments.

## 2 Materials and Methods

### 2.1 Experimental Setup

The adsorption experiments were performed using a polyvinyl chloride (PVC) column (inner diameter of 6 cm) with a packed bed of anion exchange resins (AmberLite HPR4800 OH, Dupont, USA). The resins were first rinsed with deionized (DI) water and then immersed in 0.5 M NaOH solution for at least 3 h to ensure that all the fixed groups were in the hydroxide form. Finally, the resins were rinsed with DI water before being air-dried at room temperature. 500 g of dry resins was placed inside the adsorber that gives the packed bed a height of 35 cm. During the experiment, as the resins were swelling while wetted, the height of the packed bed could reach up to 55 cm.

Air was supplied into the adsorber by a vacuum pump (LABOPORT N840.1.2FT.18, KNF, Germany). The flow rate of the pump varied between 650 and 750 mL·s^–1^ and was quantified by a mass flow meter (MASS-VIEW MV-306, Bronkhorst, The Netherlands). A heat exchanger/water-cooling unit was installed between the vacuum pump and the column to ensure a constant (room) temperature for inlet air into the column. Two CO_2_/H_2_O analyzers (LI-850, LI-COR, USA) were used to monitor the CO_2_ and H_2_O concentrations in the inlet and outlet air of the column.

**Figure 1:**
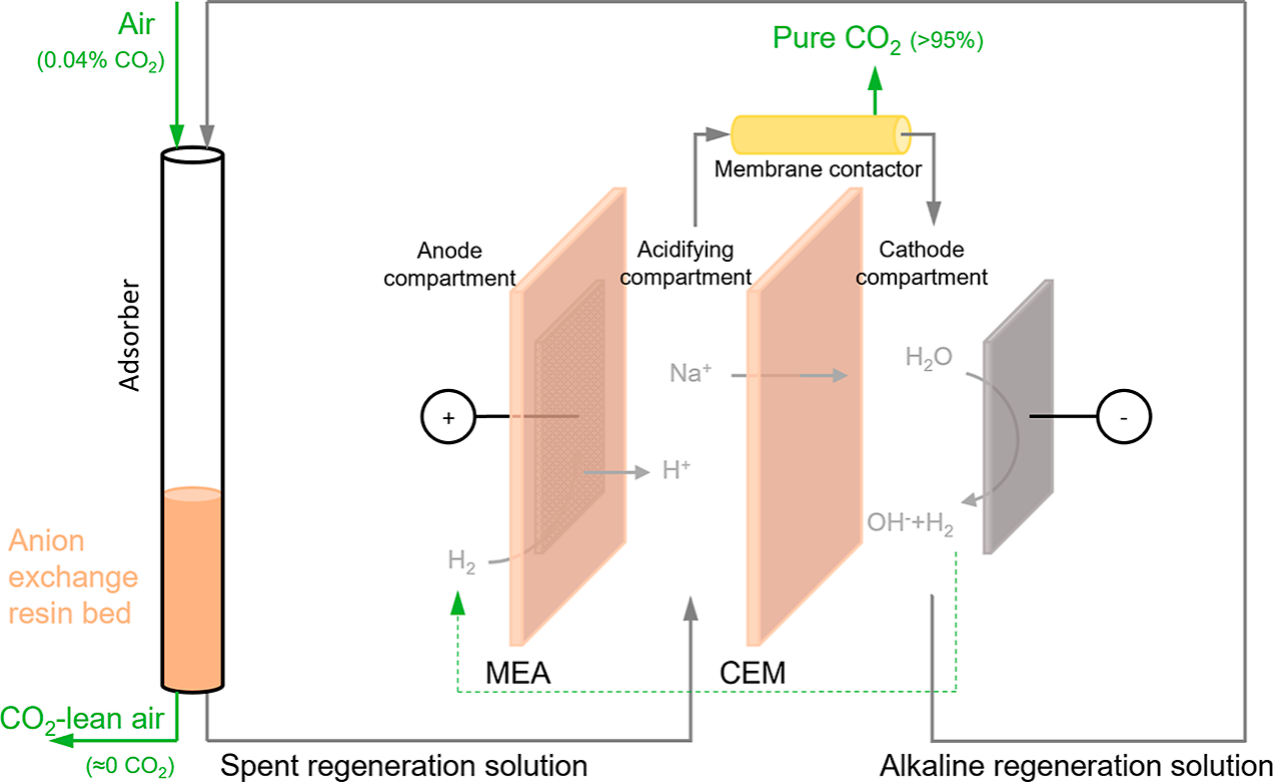
Schematic drawing of the experimental setup. Air and regeneration solution flow through the adsorber during the adsorption and desorption steps, respectively. MEA = membrane electrode assembly, CEM = cation exchange membrane.

The regeneration of the resins was achieved via an electrochemical cell directly connected with the adsorber (Figure 1). The regeneration solution was recirculated between the column and the cell with two pumps (SIMDOS 10, KNF, Germany) at a flow rate of 0.052 mL·s^–1^. The design and materials of the electrochemical cell have been described in detail in our previous work.^26^ The anode is a membrane electrode assembly (MEA) (FuelCellsEtc, TX) that comprises a platinum-coated (0.5 mg Pt·cm^–1^) gas diffusion layer (GDL) (10 cm × 10 cm) and a Nafion N117 cation exchange membrane (CEM) (15 cm × 15 cm). During operation, H_2_ gas flows into the anode compartment, where it is oxidized to H^+^ on the surface of the GDL. The produced H^+^ migrates through the CEM toward the acidifying compartment that is separated from the cathode compartment by another CEM (Nafion N117, FuelCellEtc, USA). The flow channels of the acidifying and cathode compartments are created by two polymeric (nitrile) spacers (5 × 10^–4^ cm, Sefar, Switzerland). A Ru/Ir-coated titanium mesh (9.8 cm × 9.8 cm, Magneto Special Anodes BV, The Netherlands) serves as the current collector for the anode, while a platinum-coated titanium mesh (9.8 cm × 9.8 cm, Magneto Special Anodes BV, The Netherlands) is used as the cathode. The H_2_ gas produced at the cathode can be recirculated to the anode to compensate for the H_2_ consumption, thus operating the cell in an H_2_-closed loop. However, in this study, we have used an external electrolysis cell to supply the required H_2_ at the anode to simplify the operation of the experimental setup for practical purposes. We do not expect any major change in performance of the system when hydrogen is recycled from the cathode to anode as has been demonstrated by Kuntke et al.^27^ During the operation, the spent regeneration solution was pumped into the acidifying compartment, while the effluent catholyte was recirculated back into the adsorber as the regeneration solution. The conductivities of the spent regeneration solution, acidifying solution, and catholyte were measured by three inline conductivity sensors (Memosens CLS82D, Endress+Hause, The Netherlands). The pH of the acidifying solution was measured by a pH sensor (Orbisint CPS11D, Endress+Hauser, The Netherlands). Due to the inaccuracy of the pH sensor under a high pH, the pH values of the regeneration solution and the spent regeneration solution were estimated by the OLI studio (ver. 10.0, OLI Systems, USA) based on the conductivity values of the solutions. A potentiostat (IviumStat, Ivium, The Netherlands) was used to apply the current on the cell and measure the corresponding cell voltage. The gas desorption from the acidifying solution occurred in a membrane contactor (type MM 1.7 × 8.75, 3M, USA) that provided a large surface area with hollow fiber membranes. The amount of desorbed gas was quantified by a mass flow meter (EL-FLOW Prestige FG-111B, Bronkhorst, The Netherlands), while the composition of the gas was analyzed by micro gas chromatography (μ-GC) (Varian CP-4900, Agilent, USA). The surfaces of both pristine and regenerated resins were examined by scanning electron microscopy (SEM) (JSM-6480LV, JEOL, Japan) coupled with energy dispersive X-ray spectroscopy (EDX) (NORAN Systems SIX, Thermo Fisher Scientific, USA).

Moreover, additional experiments were performed with a smaller amount of resins (8 g dry resins) in a smaller column to test the stability of the resins during chemical regeneration using a NaOH/Na_2_CO_3_ blend. The column had an inner diameter of 2.4 cm. The same vacuum pump and gas analyzers were used for the adsorption step. Each desorption step was done by using a fresh mixture of NaOH and Na_2_CO_3_ with a total Na^+^ concentration of 0.5 M and a conductivity of 58.5 mS·cm^–1^, mimicking the regeneration solution produced in the electrochemical cell in the experiments with the big column (0.192 M NaOH and 0.154 M Na_2_CO_3_). We have conducted 150 adsorption–desorption cycles, and the switch between adsorption and desorption step was controlled by three pivoted armature valves (one Type 0121 and two Type 0330, Bürkert, Germany) connected to a programmable logic controller (PLC) (LOGO! 230RC, Siemens, Germany). The ion exchange capacity (IEC) was quantified by a titration process (Supporting Information) for pristine and used resins after 68 cycles.

### 2.2 Experimental Procedure

As the resins were air-dried before placed inside the column, they needed to be regenerated to OH^–^ form before the first adsorption experiment. Initially, 1.6 L of 0.5 M NaOH solution was added to the column. The solution was then recirculated between the column and the electrochemical cell. While applying a constant current of 20 mA·cm^–2^ in the cell, the captured CO_2_ during the air-drying step was desorbed. The pretreatment process for the resins was completed when no more CO_2_ could be desorbed. Hence, the recirculation of solution and applied current were stopped. Before the first adsorption step started, the solution in the column was pumped out to be stored in an external reservoir so that it could be reused in the next desorption step. The first adsorption experiment was performed in our laboratory to quantify the CO_2_ capture capacity of the resins. The ambient conditions for this experiment were CO_2_ concentration: 394 ± 11 ppm, H_2_O concentration: 13.8 ± 1.8 mmol·mol^–1^, *T* = 25 ± 1 °C, and relative humidity (RH) = (69 ± 9)%. The following adsorption–desorption cycles were performed at two locations with different air source conditions. The first five cycles were performed using the ambient air from our laboratory: CO_2_ concentration: 427 ± 7 ppm, H_2_O concentration: 6.6 ± 0.9 mmol·mol^–1^, *T* = 25 ± 1 °C, and RH = (33 ± 5)%. The last five cycles were performed outdoors with air conditions as follows: CO_2_ concentration: 412 ± 12 ppm, H_2_O concentration: 9.6 ± 1.2 mmol·mol^–1^, *T* = 16 ± 3 °C, and RH = (84 ± 9)%. In the discussion section of the effect of humidity, “dry air” and “humid air” refer to laboratory air and outdoor air, respectively. Each adsorption step lasted for 48 h. Before the desorption step started, the stored regeneration solution in the external reservoir was added to the column. Since the resins had been dried during the adsorption step due to water evaporation, additional deionized water was added to maintain a constant total volume of solution during each desorption experiment. The amount of DI water added varied according to the different amounts of water loss in each adsorption step. Once the stored regeneration solution and the additional DI water were added to the column, the desorption step was started by recirculating the solution and applying a constant current (20 mA·cm^–2^) to the electrochemical cell. The recirculation between the column and the electrochemical cell lasted for ∼15 h, and after that, the effluent from the cell was pumped into the external reservoir (instead of the column). The desorption step was accomplished when no more solution could be pumped out of the column.

Each cycle of the experiments with the smaller column consists of 40 min of adsorption, 40 min of desorption, and 5 s of drainage. After each desorption step, the drainage step was applied to remove excess solution from the column. The air source for the adsorption steps had the following conditions: average CO_2_ concentration: 413 ± 23 ppm, H_2_O concentration: 13.7 ± 1.6 mmol·mol^–1^, *T* = 25 ± 1 °C, and RH = (68 ± 9)%. The change of CO_2_ concentration and H_2_O concentration in each cycle is reported in Figure S1.

## 3 Results and Discussion

### 3.1 Electrochemical Regeneration of AERs

**Figure 2:**
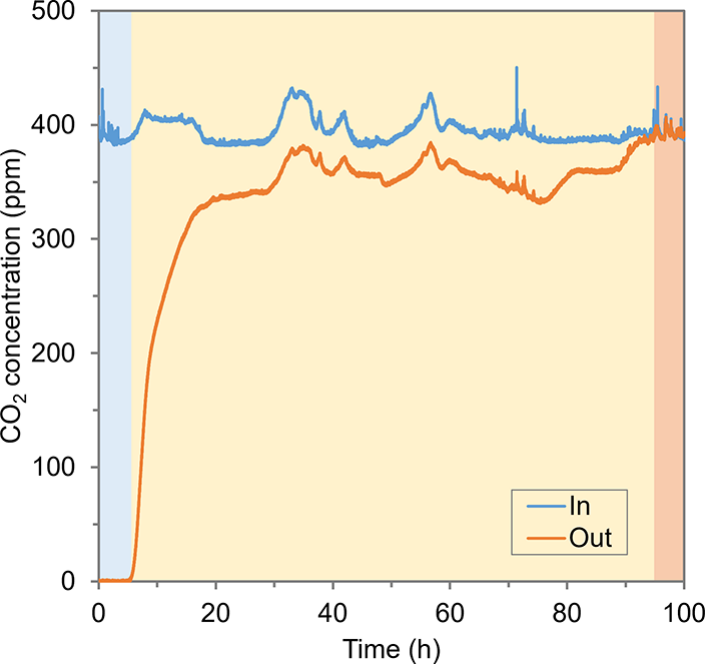
CO_2_ concentration in the influent and effluent of the adsorber during the first adsorption experiment. The adsorption step can be divided into three stages based on the adsorption rate: fast adsorption (blue), slow adsorption (yellow), and saturation (orange).

The first adsorption experiment lasted for more than 100 h, and it could be divided into three stages: fast adsorption, slow adsorption, and saturation (Figure 2). At the initial stage of the adsorption (first ∼6 h), the adsorption rate was at its maximum (0.1 mmol·g^–1^·h^–1^) as all the CO_2_ in the influent air could be fully adsorbed by the resins. With the resins becoming saturated with CO_2_, the adsorption rate decreased sharply. The adsorption rate was only 0.012 mmol·g^–1^·h^–1^ at ∼20 h, and the adsorption process remained at this low rate until ∼95 h. Finally, after 95 h, the resins were saturated with CO_2_ as the adsorption rate was 0. Within these three stages, the total amount of CO_2_ adsorbed was 0.88 mol. As the total dry mass of the resins in the column was 500 g, the CO_2_ capture capacity of the resins was 1.76 mmol·g^–1^ dry resins.

**Figure 3:**
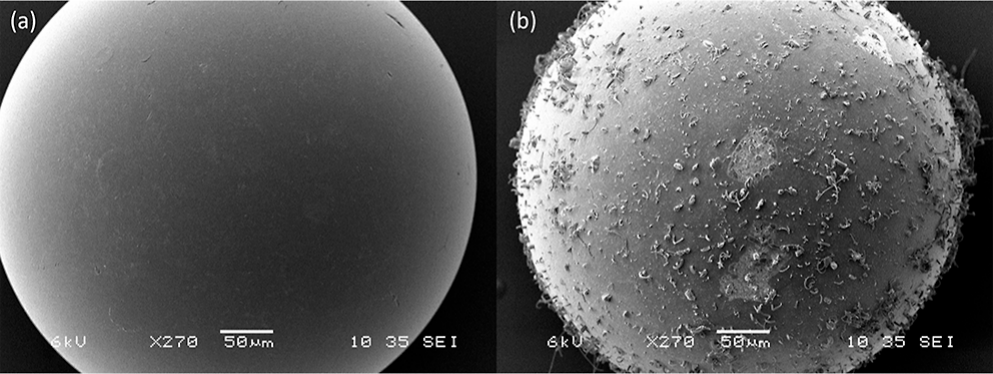
SEM images of a (a) pristine resin bead and a (b) used resin bead regenerated by the electrochemical process. The elements of the precipitation on the surface of the used resin were identified by EDX analysis as mainly Na, C, and O.

Notably, such capacity is around ten times higher than what has been reported for quaternary amine-functionalized AERs.^21^ Such a significant difference can be mainly attributed to different pretreatment and regeneration methods. In the work of Parvazinia et al., the AERs were heated up to 120 °C during the pretreatment and to 105 °C during the regeneration.^21^ However, quaternary amine-functionalized AERs in hydroxide form are not stable under such high temperatures.^28,29^ At temperatures higher than 60 °C, the occurring Hofmann degradation of the quaternary ammonium hydroxide group leads to either the loss of strong basic capacity or the loss of total ion exchange capacity.^30,31^ In contrast, both adsorption and desorption steps are at room temperature in this study. Therefore, no thermal degradation of the resins can occur, and the resins maintain a high CO_2_ capture capacity. Furthermore, the alkaline regeneration solution provided additional capacity for the subsequent adsorption step. Since the resins are regenerated with an alkaline solution, a thin layer of the solution remains on the surface of the resins after each regeneration step. As shown in Figure 3, the dry regenerated resins had precipitations on the surface that were identified as mainly Na, C, and O (see EDX results in Figure S4 and Table S1). Therefore, the residual regeneration solution (i.e., a mixture of NaOH and Na_2_CO_3_) provided extra capacity for CO_2_ capture. We have estimated the amount of CO_2_ adsorbed by the retained solution based on the following assumptions: (i) the total solution volume retained equals to the maximum water loss among all the adsorption experiments (i.e., 0.58 L), (ii) the retained solution has a composition of 0.20 M NaOH and 0.15 M Na_2_CO_3_, (iii) the alkaline solution equilibrates with 400 ppm in the air forms 0.175 M Na_2_CO_3_ and 0.150 M NaHCO_3_. Based on these assumptions, the CO_2_ adsorbed by the retained solution counts for 17.5% of the total CO_2_ capture capacity of the resin bed. To experimentally prove this estimation, we have performed one adsorption experiment with resins rinsed by de-ionized water. Without the alkaline regeneration solution on the surface, the CO_2_ capture capacity of the resins dropped by ∼17% (Table S2), which is in line with our estimation from the retained alkaline solution and composition.

After the adsorption step, CO_2_ is present on the surface of the resins in the form of CO_3_^2–^ and HCO_3_^–^. These ions are exchanged by OH^–^ during the regeneration step with the alkaline regeneration solution produced in the electrochemical cell. As a result of the ion exchange process, the pH and conductivity of the solution decrease after flowing through the resin column (Table 1). The spent regeneration solution from the outlet of the column, an aqueous solution of Na_2_CO_3_ and NaHCO_3_, is fed into the electrochemical cell. In the electrochemical cell, the solution is first acidified so that CO_2_ can be desorbed into the gas phase. The desorbed gas was under atmospheric pressure and confirmed with μ-GC to contain more than 96% of CO_2_ (detailed composition is shown in Table S3). Then, the OH^–^ produced in the hydrogen evolution reaction (HER, cathode compartment) increased the pH of the CO_2_ depleted regeneration solution. The regeneration of the resins was considered completed when the conductivities of the regeneration solution at the outlet and inlet of the adsorber were equal. Meanwhile, the gas desorption rate decreased to zero since a negligible concentration of CO_3_^2–^ or HCO_3_^–^ was present in the feed solution of the electrochemical cell (Figure S6).

**Table 1:** pH and Conductivity Values of Regeneration Solution, Spent Regeneration Solution, and Acidifying Solution^a^

	pH	conductivity (mS/cm)
regeneration solution (adsorber inlet)	13.0^b^	53.4 ± 3.6
spent regeneration solution (adsorber outlet)	10.0^b^	31.2 ± 1.1
acidifying solution	6.5 ± 0.2	12.0 ± 0.7

a All values represent the average and standard deviation of five desorption steps.

b Not measured due to inaccuracy of pH sensors under high pH, but estimated by OLI Studio based on the average conductivity and Na^+^ concentration of the solution.

**Figure 4:**
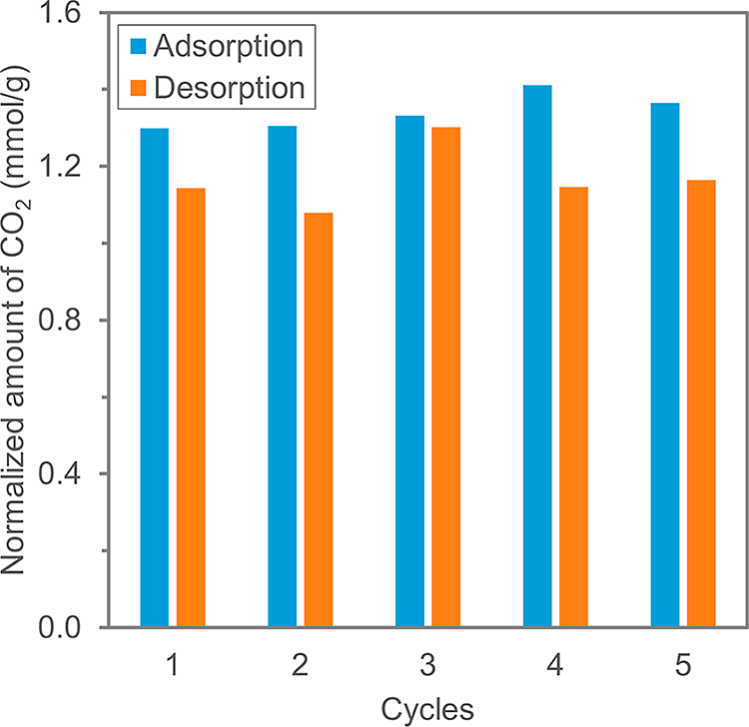
Experimental results from five repeated adsorption–desorption cycles showing the normalized amount of CO_2_ adsorbed and desorbed per gram of dry resins.

The CO_2_ capture capacity of the resins was restored after they were regenerated to the OH^–^ form. We have repeated the adsorption–desorption cycle five times using lab air. The complete DAC system showed a stable performance over five lab cycles regarding the amount of CO_2_ adsorbed and desorbed (Figure 4). Instead of reaching the full CO_2_ capture capacity of the resins with ∼95 h of adsorption, these adsorption steps were limited to 48 h, when more than 75% of the adsorption was accomplished. The normalized amount of CO_2_ adsorbed in five cycles was 1.34 ± 0.05 mmol·g^–1^, while 1.17 ± 0.08 mmol·g^–1^ was desorbed. The discrepancy between the adsorption and desorption could be attributed to (i) the wetting of the resins during regeneration and/or (ii) CO_2_ leakage in the setup during desorption. The resins are wetted by the regeneration solution at the beginning of a desorption step. According to Wang et al., CO_2_ could be desorbed from dry quaternary amine-functionalized resins when the resins are wetted.^23^ Therefore, part of the adsorbed CO_2_ could escape from the adsorber before the ion exchange occurs. Moreover, a small amount of CO_2_ could leak from the electrochemical system during desorption as already observed in our previous work.^26^ Despite the leakage of CO_2_, the resins could be sufficiently regenerated during the desorption steps as a consistent amount of adsorption was observed during consecutive adsorption steps.

### 3.2 Stability of the AERs

**Figure 5:**
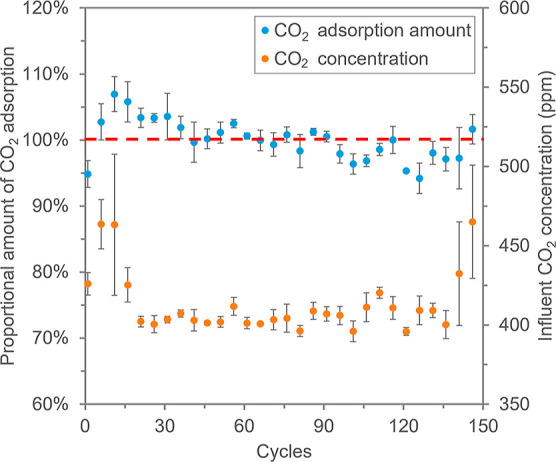
Proportional change of CO_2_ adsorption amount in 150 adsorption–desorption cycles and the influent CO_2_ concentration in these cycles. The average CO_2_ adsorption amount of all the 150 cycles was considered as 100% and then the proportional CO_2_ adsorption amount of each cycle was calculated accordingly. Symbols: average value of every five consecutive cycles; error bars: standard deviations within the five cycles.

The application of a sorbent for carbon capture requires high performance stability over long-term usage. Previous studies on the degradation of the resins used for carbon capture mainly focused on the thermal stability of the resins.^21,24^ Nevertheless, the process proposed in this work is operated under ambient/room temperature, so thermal degradation is not expected. On the other hand, the stability of the resins could be affected by repeated dry–wet cycles. The exposure of AERs under dry–wet cycles can change the smoothness of the resin surface (Figure S5) and cause the desorption of ions due to the shrinking of the resin beads.^32^ However, to the best of our knowledge, no evidence has been found that ion exchange capacity (IEC) of resins would change during dry–wet cycles. Therefore, 150 adsorption (dry)–desorption (wet) cycles were conducted to investigate the stability of the HPR4800 AERs for carbon capture. Figure 5 has depicted the change of the CO_2_ adsorption amount over these 150 cycles and the according influent CO_2_ concentration.

Overall, the proportional change of CO_2_ adsorption amount of the last five cycles was about 100%, which indicates no measurable loss of the resin performance. However, there was some fluctuation of the CO_2_ adsorption amount over the 150 cycles. In the first 10 and the last 10 cycles, the amount of CO_2_ been adsorbed increased. This increase was mainly attributed to the rise in CO_2_ concentration in the influent (with a correlation coefficient between the amount of CO_2_ adsorbed vs. CO_2_ concentration in the influent of 0.98 and 0.84 for the first and last ten cycles, respectively). From cycle 11 to cycle 140, the amount of CO_2_ been adsorbed showed a slightly decreasing trend over the cycles. Meanwhile, we have also noticed an 11% decrease in the flow rate of the air supply pump from cycle 1 to cycle 150 (Figure S2). The flow rate of the influent air can affect the amount of CO_2_ adsorption from two perspectives. First, as the adsorption time was kept constant in the experiments, a lower influent flow rate means a lower total amount of CO_2_ fed to the resins. Second, a lower flow velocity in the column resulted in a higher adsorption efficiency due to less limitation from diffusion and reaction kinetics.^33^ Thus, the reduction of the adsorption amount was not caused by the degradation of resins but mainly due to the slightly lower flow rate of the air supply pump over 150 cycles. We corrected the proportional change of CO_2_ adsorption assuming a constant flow rate and constant CO_2_ concentration in the influent, and the results are plotted in Figure S3. Finally, the CO_2_ adsorption is attributed to the quaternary amine groups on the resins that could be quantified by IEC. The IEC of the pristine resins was 1.85 ± 0.01 meq·g^–1^, while the IEC remained constant to be 1.86 ± 0.06 meq·g^–1^ after 68 cycles. Therefore, regardless of the impact of influent CO_2_ concentration and air flow rate, the resins showed stable adsorption performance.

### 3.3 Effect of Air Humidity

After the regeneration step, the resins are swelled (water retention capacity: 58–74%) and surrounded by alkaline solutions. As stated before, one of the advantages of regenerating the resins with an alkaline solution is that the remaining NaOH and Na_2_CO_3_ on the surface of the resins provide extra capacity for CO_2_ capture. However, the water on the surface of the resins and inside the resins evaporates during adsorption when air flows through the resins. The total amount of water evaporated during the adsorption step is counted as water loss of the process and needs to be compensated by adding the corresponding amount of deionized water before the regeneration step. Since water evaporation is influenced by the humidity of the air, we have performed adsorption tests with influent air under different humidity conditions, calculating the water loss during adsorption based on the concentration difference of H_2_O in the inlet/outlet air of the adsorber. The comparison between the performance of the system with dry air and humid air is shown in Table 2.

**Table 2:** Comparison of the Amount of CO_2_ Adsorption and Specific Water Loss between Adsorption Experiments Using Dry Air (RH = 33%) and Humid Air (RH = 84%)^a^

	dry air	humid air
CO_2_ adsorption (mmol CO_2_/g dry resins)	1.34 ± 0.05	1.04 ± 0.04
water loss (g H_2_O/g CO_2_)	18.01 ± 1.59	6.65 ± 2.96

a All values represent the average and standard deviation of five adsorption–desorption cycles using different sources of air.

A higher relative humidity caused a significant reduction of water loss by evaporation in the experiments. However, using air sources with higher humidity also leads to lower CO_2_ capture capacity. According to Wang et al., the adsorption of CO_2_ on quaternary amine-functionalized resins involves the equilibrium between bicarbonate state and carbonate state (eq 3).^23^ When the resins are wet, they are mainly in the carbonate state; thus, two active sites of the quaternary amine group can capture one CO_2_ molecule in the form of bicarbonate. When the resins dry, the CO_2_–H_2_O equilibrium shifts to the bicarbonate state where the ratio between quaternary amine and CO_2_ becomes 1:1, implying a higher CO_2_ capture capacity. As a result, there is a trade-off between the CO_2_ capture capacity of resins and water loss in the process.
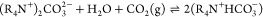
3

The maximum water retention capacity of the resins is 74%. If all retained water is evaporated during the adsorption experiment and given a CO_2_ adsorption amount of 1.34 mmol/g (average from dry air experiments), the maximum specific water loss was 12.50 g H_2_O/g CO_2_. The water loss during the experiments with dry air was higher than this maximum value, which indicated the operation of the experiments could be optimized to reduce the water loss. Moreover, one of the state-of-the-art technologies using liquid alkaline absorbent for DAC consumes 4.32 g H_2_O/g CO_2_ captured at ambient conditions of 20 °C and 64% relative humidity.^34^ Therefore, the experiments with humid air in this study already showed an average water loss in the same range as state-of-the-art. Finally, other technologies (e.g., condensation) could be combined with the current adsorption step to retrieve the evaporated water.

### 3.4 Outlook and Perspectives

The feasibility of a DAC system requires a low energy consumption, long lifetime of the sorbents, and low negative environmental impacts.^35^ The energy consumption of the proposed technology mainly comes from the mechanical energy to overcome the pressure drop in the adsorber during adsorption and the electrical energy consumed by the electrochemical cell during desorption.

The mechanical energy required to supply the air through the adsorber is proportional to the pressure drop of the column. The pressure drop (Δ*p*, Pa) can be estimated by Ergun equation^36^
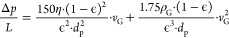
4where *L* is the height of the packed bed (m), η is the dynamic viscosity of the fluid (Pa·s), ε is the void fraction of the packing, *d*_p_ is the resin diameter (m), *v*_G_ is the channel velocity of the air inside the column (m·s^–1^), and ρ_G_ is the fluid density of air (kg·m^–3^). The pressure drop over the adsorber in this work was around 5000 ± 1000 Pa at *v*_G_ = 20 cm·s^–1^ (where the variation was due to changing water content of the resins during adsorption). However, while the design of the adsorber has been out of the scope of the present work, the bed height was not optimized (varying between 35 and 55 cm depending on the water content). As shown in eq 4, the pressure drop increases linearly with the height of the packed bed. Thus, a shorter packed bed could be applied in future applications of the technology to achieve lower pressure drops. For instance, Yu and Brilman studied a packed bed of ion exchange resins with a height of 1 cm.^33^ In their work, the mechanical energy for air supply could be as low as 26.4 kJ·mol^–1^ CO_2_ captured with a pressure drop of 118.4 Pa. Yu and Brilman have also proposed a radial flow contactor that showed a low energy consumption and a short adsorption time.^37^ Moreover, further studies should investigate the pressure drop in the adsorber filled with wet resins. The void fraction of the packing ε is expected to decrease as the void would be partly filled with regeneration solution, which would increase the pressure drop. On the other hand, the resin diameter *d*_p_ increases when the resins are swelled with liquid, which would lead to a decrease in pressure drop. Therefore, the pressure drop profile during one complete adsorption experiment needs to be studied in an optimally designed adsorber.

The average electrical energy consumption of the five desorption experiments in the laboratory was 537 ± 33 kJ·mol^–1^ under 20 mA·cm^–2^. The electrochemical cell used in this study is not optimized as the electrode overpotentials of the cell count for approximately half of the energy consumption. We have achieved 374 kJ·mol^–1^ under 5 mA·cm^–2^ in our previous work, and the theoretical minimum energy consumption of the desorption step was 164 kJ·mol^–1^.^26^ Higher current density resulted in higher electrode overpotentials. Therefore, future research should focus on developing strategies to reduce electrode overpotentials under high current density. Moreover, the adsorption step with AERs could potentially be combined with other electrochemical processes that can create a pH swing. For instance, Eisaman et al. proposed a bipolar membrane electrodialysis (BPMED) process for carbon capture, where a pH swing was built in two adjacent compartments alongside a bipolar membrane.^38^ While combining with CO_2_ capture using AERs, the low pH compartment could desorb high purity CO_2_ gas, and the high pH compartment could reproduce the regeneration solution for the resins.

Finally, despite that the energy consumption of the system still needs to be optimized, the combined DAC process with AERs and the electrochemical regeneration has shown the potential to be further developed. The resins have a high CO_2_ capture capacity of 1.76 mmol·g^–1^ dry resins, and they are stable in 150 adsorption–desorption cycles. The sorbents need to last for tens of thousands of cycles to make the DAC process economically feasible,^39^ so more studies should be conducted on the stability of the resins. However, due to the room-temperature operation of our combined DAC process, we believe the resins could outlast other thermal-regenerated sorbents. Furthermore, other solid sorbents can be investigated for the feasibility of electrochemical regeneration. Sorbents with high CO_2_ capture capacity, high chemical stability, and low carbon footprint are necessary for practical application of the combination with the electrochemical system.^40^ Lastly, a techno-economic analysis would help find the optimal location for implementing such a process, as the local weather conditions (e.g., relative humidity) have a considerable impact on the capacity of the resins and the water loss of the process.

## Supporting Information Available

Ion exchange capacity measurement methods; influent conditions for the 150 adsorption–desorption cycles; SEM and EDX analysis of the resin surface; results of an adsorption experiment with DI-water rinsed resins; composition of the gas desorbed from the electrochemical cell; change of measured parameters in a desorption step (PDF)

## Notes

The data underlying this study are openly available in 4TU.ResearchData at https://doi.org/10.4121/19180913.v1.
