# Supplementary material for: Direct Air Capture Using Electrochemically Regenerated Anion Exchange Resins
Source: Environ Sci Technol. 2022 Aug 4;56(16):11559–66. doi: 10.1021/acs.est.2c01944 (PMC9386902; doi:10.1021/acs.est.2c01944)
Supplement: Supplementary file 1 — es2c01944_si_001.pdf [file es2c01944_si_001.pdf]

Supporting Information

Direct air capture using electrochemically regenerated anion exchange resins

Qingdian Shu[a,b], *Marina Haug*[a,c], *Michele Tedesco*[a], *Philipp Kuntke*[*,a,b], *Hubertus V. M. Hamelers*[a,b]

[a] Wetsus, European Centre of Excellence for Sustainable Water Technology, Oostergoweg 9, 8911MA, Leeuwarden, the Netherlands

[b] Environmental Technology, Wageningen University, Bornse Weilanden 9, 6708WG, Wageningen, the Netherlands

[c] Faculty of Natural and Environmental Sciences, Hochschule Zittau/Görlitz, Theodor-Körner-Allee 16, 02763, Zittau, Germany

*Corresponding author: Philipp Kuntke, Department of Environmental Technology, Wageningen University, Bornse Weilanden 9, P.O. Box 17, 6700AA Wageningen, the Netherlands; Phone: +31-58-2843000; email: Philipp.Kuntke@wur.nl

Number of pages: 10

Number of Tables: 3

Number of Figures: 6

S.1. Ion exchange capacity measurement methods

The ion exchange capacity (IEC) of the resins was quantified by a titration process. The resins (fresh or used) were rinsed with de-ionized (DI) water and then air-dried. Approximately 2 grams of the dry resins were taken for each titration. The resins were first immersed in 200 mL 1 mol/L NaOH for more than 12 hours (with stirring) so that all the resins should be in OH^- form. Afterwards, the resins were rinsed with DI water. Excess DI water was removed by filtration. The solid resin particles were transported to 50 mL 0.1 mol/L NaCl solution and immersed in the solution for more than 12 hours with stirring. The concentrations of Na^+ and Cl^- were measured by ion chromatography (761 Compact IC, Metrohm, Switzerland). The IEC of the resins is calculated by:

$$IEC = \frac{([Cl^-]_0 - [Cl^-]_1/([Na^+]_1/[Na^+]_0)) \times V}{M} \quad \text{(S1)}$$

where $[Cl^-]_0$ and $[Na^+]_0$ are the concentrations of Cl^- and Na^+ in the fresh prepared 0.1 mol/L NaCl solution (in mol·L^{-1}), $[Cl^-]_1$ and $[Na^+]_1$ are the concentrations of Cl^- and Na^+ in the NaCl solution where the resins were immersed for more than 12 hours (in mol·L^{-1}), V is the volume of the NaCl solution (in L), and M is the weight of dry resins used for the titration (in g).

S.2. Influent conditions for the 150 adsorption-desorption cycles

150 adsorption-desorption cycles were performed in a small column. During the adsorption steps, the CO_2 concentration and H_2O concentration in the air influent varies

over time. The change of average influent CO_2 concentration and H_2O concentration in each cycle is plotted in Figure S1.

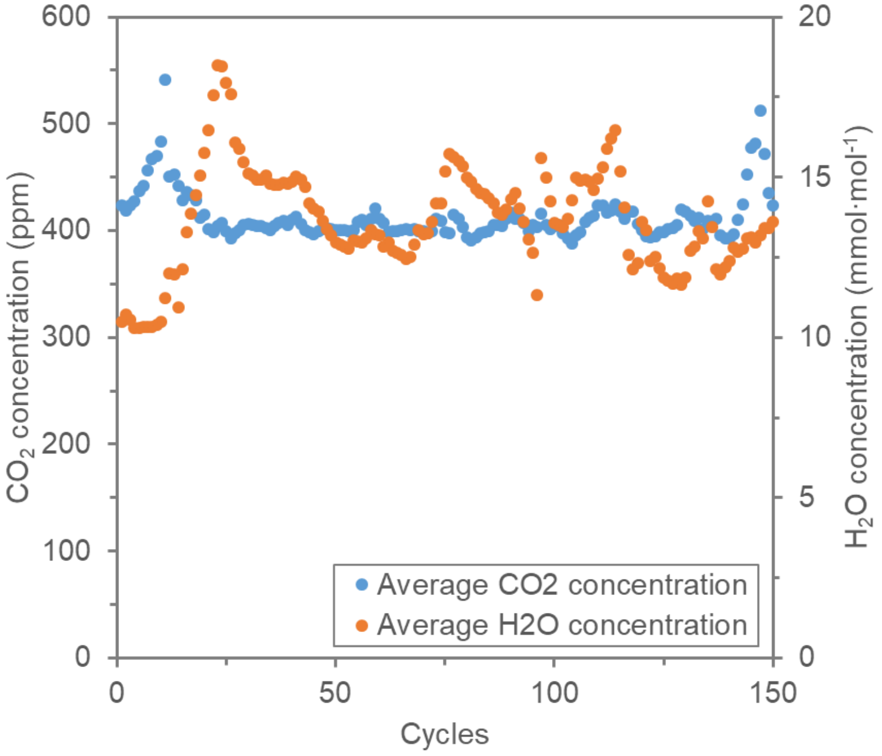

Figure S1. Average influent CO_2 concentration and H_2O concentration in the 150 adsorption-desorption cycles.

The flow rate of air influent in the 150 adsorption steps is depicted in Figure S2. Not all the cycles were measured due to practical limitations (e.g., overnight running of experiments). The flow rates of the cycles not measured were assumed to be the same as the last measured cycle before, for instance, the flow rate of cycle 2 to cycle 18 was assumed the same as the flow rate of cycle 1. Overall, the flow rate of the air influent decreased 11% from cycle 1 to 150.

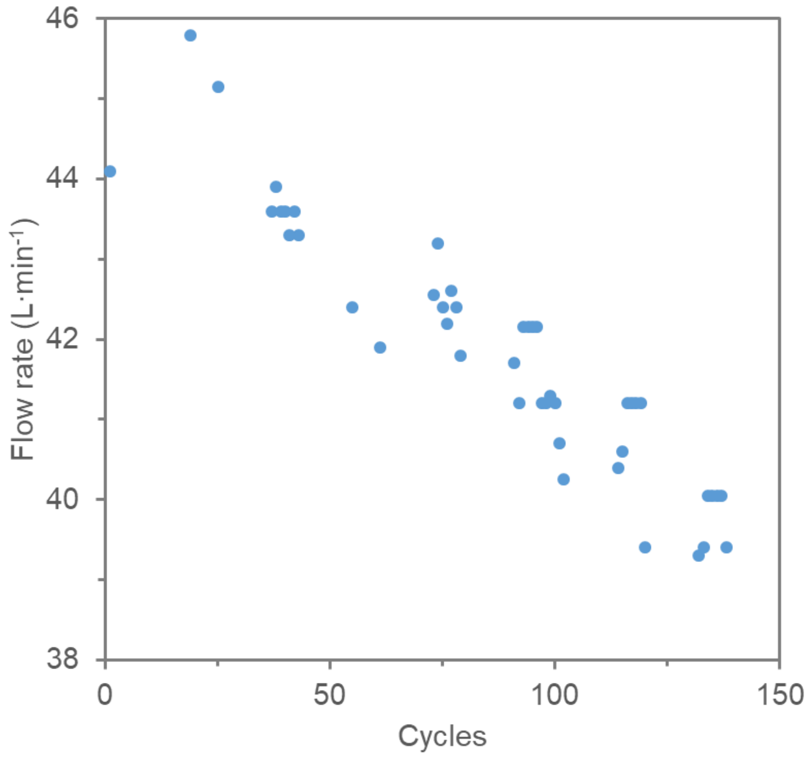

Figure S2. The flow rate of air influent during the 150 adsorption-desorption cycles

We have also corrected the proportional change of CO_2 adsorption amount based on a constant influent CO_2 concentration and a constant influent flow rate (Figure S3).

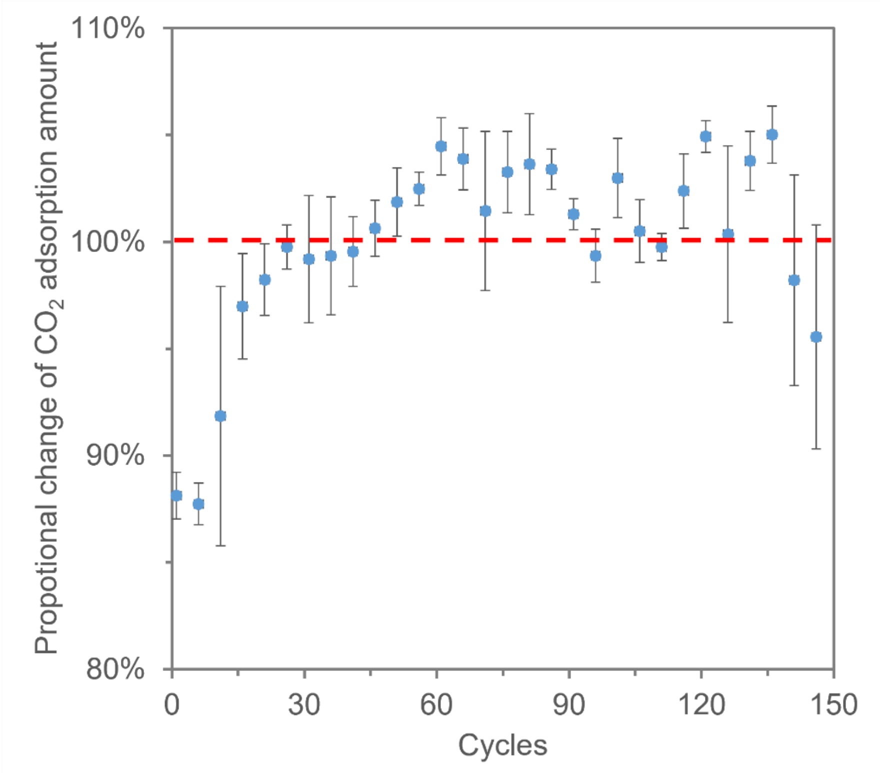

Figure S3. Proportional change of CO_2 adsorption amount in 150 adsorption-desorption cycles corrected by influent CO_2 concentration and flow rate.

S.3. Scanning electron microscope (SEM) and energy dispersive X-Ray spectroscopy (EDX) analysis of resin surface

The scanning electron microscope (SEM) and energy dispersive X-Ray spectroscopy (EDX) images of the surface of a used resin bead are shown in Figure S4. The weight percentage of all the elements detected are listed in Table S1. The precipitation on the surface of the resin was identified to contain mainly Na, C, and O.

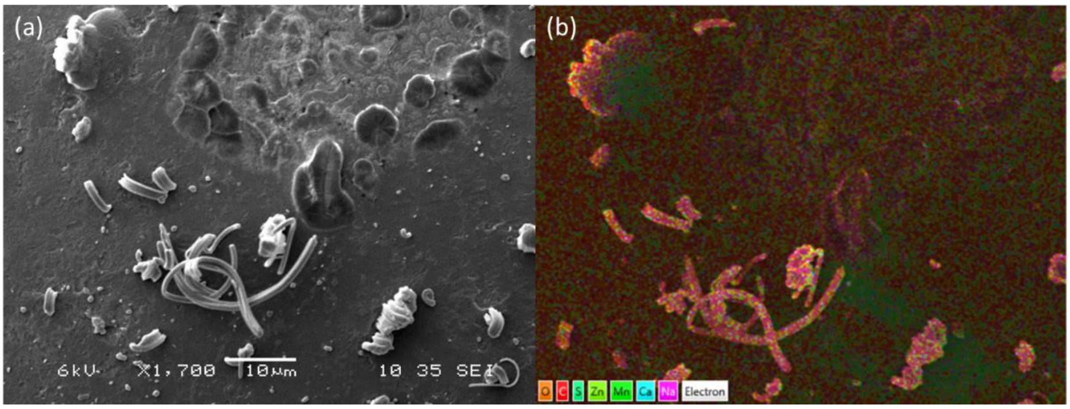

Figure S4. (a) Scanning electron microscope (SEM) and (b) energy dispersive X-Ray spectroscopy (EDX) images of the surface of a used resin bead.

Table S1. Weight percentage of the elements detected by energy dispersive X-Ray spectroscopy (EDX) analysis on the surface of a used resin bead.

Element	Wt%
C	79.84
O	16.77
Na	3.36
Al	0.01
Si	0.02
Total:	100.00

After rinsing the used resin with de-ionized water, the surface of the resin revealed to be rougher than the surface of a fresh resin (Figure S5).

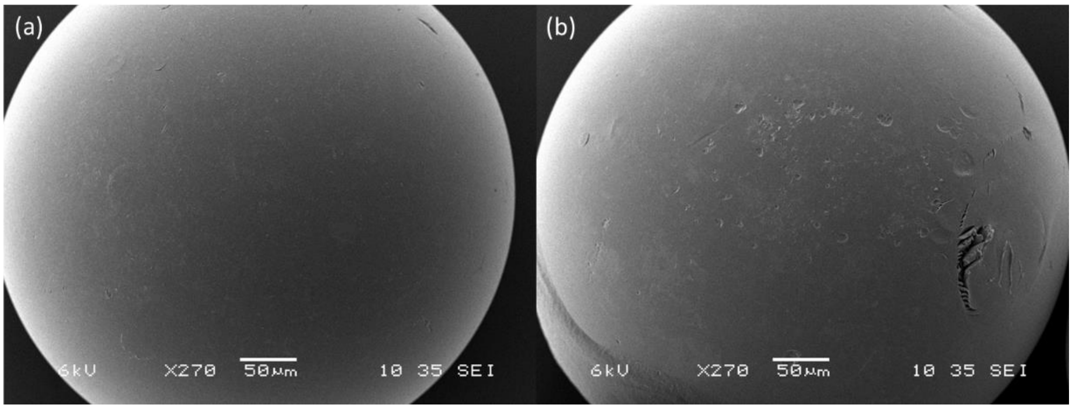

Figure S5. SEM image of (a) a fresh resin and (b) a used resin (rinsed by de-ionized water).

S.4. Adsorption with DI-water rinsed resins

After each regeneration step, a layer of the alkaline regeneration solution remains on the surface of the resins. We have performed one adsorption experiment with DI water-rinsed resins so that the influence of the remaining regeneration solution on the adsorption performance could be identified. Table S2 showed the comparison of the CO_2 adsorption amount of the rinsed column with one of the non-rinsed column experiment. The effect of air humidity is excluded in this comparison as both experiments have a similar water loss. Therefore, the CO_2 adsorption amount decreased by 17% without the remaining regeneration solution.

Table S2. Comparison of CO_2 adsorption amount and water loss of non-rinsed and DI water-rinsed resin column.

	Non-rinsed column	DI water-rinsed column
Adsorption amount (mmol/g)	1.08	0.90
Water loss (L)	0.27	0.27

S.5. Composition of the gas desorbed from the electrochemical cell

The gas composition of the gas desorbed from the electrochemical cell is listed in Table S3. In all the experiments, the gas desorbed contains more than 96% of CO_2. The H_2 gas is likely permeated from the anode through the MEA to be present in acidifying solution and desorbed into gas phase, while the presence of O_2 and N_2 is most likely caused by the introduction of air during sampling.

Table S3. Gas composition of the desorbed gas from the electrochemical cell during 10 desorption experiments.

	CO_2	H_2	O_2	N_2
Experiment 1	97.80%	0.83%	0	1.37%
Experiment 2	96.21%	1.02%	0.64%	2.13%
Experiment 3	96.54%	0.78%	0.62%	2.06%
Experiment 4	96.95%	0.83%	0.59%	1.63%
Experiment 5	96.94%	0.81%	0.59%	1.66%
Experiment 6	96.44%	1.11%	0.65%	1.80%
Experiment 7	96.36%	1.07%	0.68%	1.89%
Experiment 8	96.77%	0.95%	0.61%	1.67%
Experiment 9	96.23%	1.17%	0.69%	1.91%
Experiment 10	97.82%	0.97%	0	1.21%

S.6. Change of measured parameters in a desorption step

During the desorption step, the pH of the acidifying solution decreased so that CO_2 could be desorbed into the gas phase (Figure S6a). Due to the ion exchange between OH^- and CO_3^{2-}/HCO_3^-, the conductivity of the outlet solution of the adsorber is lower than the conductivity of the inlet solution (Figure S6b). The desorption step finishes when the conductivities of the outlet and inlet solution of the adsorber are equal.

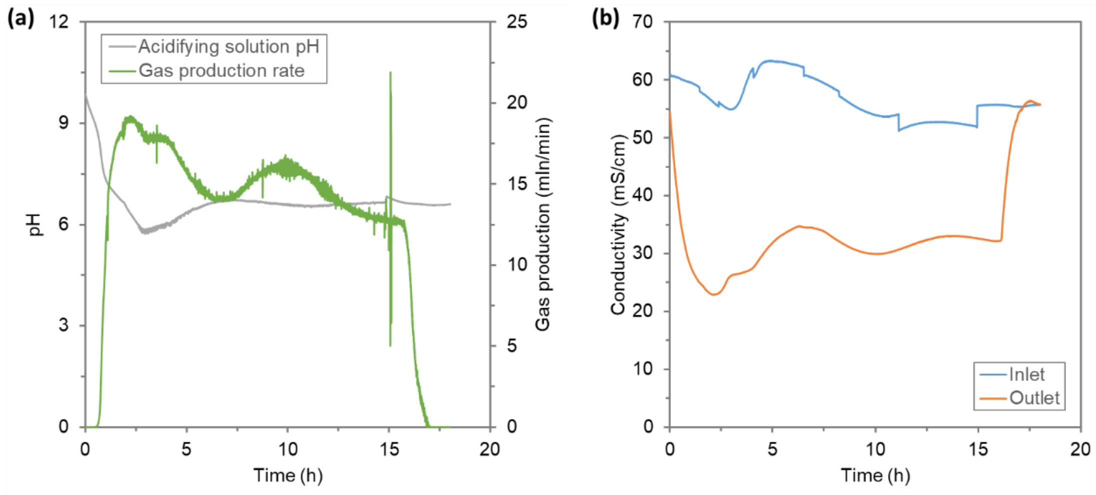

Figure S6. (a) The pH of acidifying solution and gas desorption rate change over time in a desorption step; (b) change of conductivity of the inlet and outlet solution of the adsorber over time during a desorption step.
